# Effectiveness of the Osaka Metropolitan University Hospital Eating and Swallowing Manual for Dysphagia Triage in Patients at Admission: A Retrospective, Comparative Cross-Sectional Study

**DOI:** 10.7759/cureus.83626

**Published:** 2025-05-07

**Authors:** Mitsuhiko Ikebuchi, Tamotsu Nakatsuchi, Takeshi Yamauchi, Yoichi Ohta, Yukihide Minoda, Hidetomi Terai, Hirokazu Sakamoto, Masaoshi Kawashima, Keio Ookubo, Haruna Okamura

**Affiliations:** 1 Department of Rehabilitation Medicine, Osaka Metropolitan University Graduate School of Medicine, Osaka, JPN; 2 Department of Rehabilitation Medicine, Tsuji-Geka Rehabilitation Hospital, Osaka, JPN; 3 Department of Medical Education and General Medicine, Osaka Metropolitan University Graduate School of Medicine, Osaka, JPN; 4 Department of Orthopedic Surgery, Osaka Metropolitan University Graduate School of Medicine, Osaka, JPN; 5 Department of Otolaryngology, Osaka Metropolitan University, Osaka, JPN; 6 Department of Rehabilitation, Osaka Metropolitan University Hospital, Osaka, JPN; 7 Department of Nursing, Osaka Metropolitan University Hospital, Osaka, JPN; 8 Department of Nutrition, Osaka Metropolitan University Hospital, Osaka, JPN

**Keywords:** aspiration pneumonia, dysphagia triage, eating and swallowing team, eating assessment tool-10 (eat-10), omues manual, repetitive saliva swallowing test (rsst)

## Abstract

Background

Aspiration pneumonia is a potentially fatal condition that is more commonly seen in older people and has a high mortality rate. As the world population is aging, aspiration pneumonia may likely become more common worldwide, including Japan and the United States. Although aspiration pneumonia is regarded as a preventable condition, its prevention is attracting a great deal of attention, with many published studies reporting screening tests.

We developed the Osaka Metropolitan University Hospital Eating and Swallowing (OMUES) manual for dysphagia triage in all hospitalized patients to prevent aspiration pneumonia.

In this study, we investigated the effectiveness of the OMUES manual for dysphagia triage in hospitalized patients by assessing changes in the incidence of aspiration pneumonia after introducing the OMUES manual.

Materials and method

The OMUES manual is designed for use by nurses without didactic training. Inpatients who meet the criteria for using the OMUES manual undergo a swallowing function assessment in three stages: the Eating Assessment Tool-10 (EAT-10) (stage 1), the Repetitive Saliva Swallowing Test (RSST) (stage 2), and the eating scene observation (stage 3).

The study participants included 12,395 patients (6,000 males and 6,395 females) who were admitted between September 1, 2021, and August 31, 2022, excluding minors, patients with aspiration pneumonia on admission, and patients on emergency hospitalization, whose data were retrospectively extracted from the electronic medical records. These patients were examined in two groups: before and after introducing the OMUES manual. We closely examined patients with aspiration pneumonia to identify the cause and compared these between the two groups. Furthermore, as a secondary analysis, we also evaluated the implementation status of the OMUES manual.

Results

Before introducing the OMUES manual, there were 6,546 patients (3,164 males/3,382 females) and 52 patients with aspiration pneumonia (0.8%). After introducing the OMUES manual, the number of patients was 5,849 (2,836 males/3,013 females) and 42 cases (0.7%) with aspiration pneumonia. No statistically significant difference was observed between the two groups. As for the causes of aspiration pneumonia, factors associated with poor arousal were significantly reduced after introducing the OMUES manual, but the number of iatrogenic cases remained unchanged (21/52 cases [40.4%] and 19/42 cases [45.2%] before and after introducing the OMUES manual, respectively). The majority were associated with upper gastrointestinal endoscopy. The utilization rate was 92.5% in stage 1 of the OMUES manual, 50.9% in stage 2, and 60.0% in stage 3. The OMUES manual utilization rate, calculated by multiplying the utilization rates at each of stages 1, 2, and 3, was 28.3%.

Conclusions

We investigated the effectiveness of using the OMUES manual for dysphagia triage on all hospitalized patients. Although there was no statistically significant difference in the incidence of aspiration pneumonia before and after introducing the OMUES manual, our results suggest that it is contributing to its prevention. In addition, endoscopic examination revealed cases of aspiration pneumonia even in patients with normal swallowing function, and further investigation is required.

## Introduction

Aspiration pneumonia is more common in older people and is regarded as a highly malignant form of pneumonia. As the world population is aging, aspiration pneumonia may likely become more common worldwide. Global trends in aspiration pneumonia are lacking, including in the WHO database [1]. However, evidence exists in reports from the United States and Japan. In the United States, Gupte et al. investigated data on the causes of deaths registered at a disease management and prevention center between 1999 and 2017 and reported that the mean number of deaths from aspiration pneumonia was 58,576 per year, equivalent to 21.85 per 100,000 population. They also found that 76.0% of patients who had died were older people aged ≥75 years and that neurological disease, upper gastrointestinal disease, lung disease, and sedative-related disorders were frequently associated with death from aspiration pneumonia [2]. In Japan, the number of deaths from aspiration pneumonia has also been steadily rising. According to the FY 2022 Ministry of Health, Labor, and Welfare Vital Statistics, the total number of deaths from aspiration pneumonia was 56,068 in 2022, equivalent to 45.9 per 100,000 population, making it the sixth most common cause of death [3].

However, aspiration pneumonia is regarded as a preventable disease, and numerous screening tests designed for its prevention focused on evaluating eating and swallowing function have been reported. Many of the studies reporting these screening tests focused on patients with cerebral stroke, for whom screening tests are reportedly effective [4-7]. In all these reports, assessments involved observing patients while they swallowed a small amount of water or a small amount of food with a small amount of water and were conducted by nurses who had undergone didactic training. According to a meta-analysis by Boaden et al., tests were conducted by nurses in 21 of the 25 included studies [8].

There are several reports on swallowing screening tests for patients other than those in stroke wards. Cichero et al. performed an intake swallowing screening test on 442 inpatients in a 982 beds general tertiary teaching hospital general ward by nurses with specialized training and reported that the screening test was useful for dysphagia triage and that education regarding swallowing disorders is necessary [9]. Taveira et al. introduced an eating and swallowing screening test conducted by nurses with specialized training in a medical ward that handles 700 inpatients a year, investigated and compared 128 patients before and 125 after the introduction, and reported a statistically significant decrease in the number of respiratory complications [10]. In Japan, Kitamura et al. reported that as a preliminary step to screening all inpatients at a university hospital with approximately 1,100 beds, nurses with specialized training conducted screenings on all 1,335 inpatients in five wards and reported the possibility of preventing choking accidents and aspiration pneumonia [11].

Because Osaka Metropolitan University Hospital is a university hospital with approximately 900 beds, its staff deals with patients with a wide range of diseases, including intractable diseases, and the hospital admits an average of approximately 80 patients a day, including tertiary emergency patients. As it is a teaching institution, there is a high rate of turnover of doctors and nurses within a few years, and the number of turnovers has increased due to the COVID-19 pandemic, making it unrealistic for swallowing function evaluations to be conducted by specialists, such as speech therapists (STs), or by medical professionals who have undergone specialist training.

Before 2022, our hospital had different countermeasures for each department and ward. However, due to the growing awareness of aspiration pneumonia in Japan, there was an urgent need to develop a unified manual to help prevent aspiration pneumonia by triaging dysphagia, which is one of the causes of aspiration pneumonia, in hospitalized patients and initiating early professional intervention.

The Eating and Swallowing Team (EST) at our hospital, comprising members from the Departments of Rehabilitation, Otorhinolaryngology, and Dentistry, as well as STs, nutritionists, and certified dysphagia nursing nurses, developed the Osaka Metropolitan University Hospital Eating and Swallowing (OMUES) Manual. The manual was developed for dysphagia triage to help prevent aspiration pneumonia in all inpatients, and was designed to be usable even by nurses who have not undergone didactic training was required. The EST developed the OMUES manual based on its own original contributions, while drawing upon the work of Kitamura et al. [11], whose study was conducted at a university hospital of comparable size in the same region as our institution. We conducted a three-month trial implementation of OMUES Manual to 74 patients in one ward under the supervision of Department of Quality and Safety Management and Department of Nursing, and it was determined that there were no operational or safety issues.

In this study, we investigated the effectiveness of the OMUES manual for dysphagia triage on hospitalized patients by investigating changes in the incidence of aspiration pneumonia after introducing the OMUES manual.

## Materials and methods

Design

This study is a retrospective, comparative cross-sectional study conducted by using hospital records.

OMUES manual

The OMUES manual is designed for use by nurses without didactic training who have to deal with patients with a wide range of conditions, including intractable diseases, but have used the written procedures attached to the manual for self-study. The current version covers patients who do not fall under the exclusion criteria but who meet the introduction criteria shown (Table 1). Minors under the age of 15, patients who were unable to answer questions on their own, and patients who were admitted to the hospital in an emergency were excluded.

**Table 1:** Inclusion and exclusion criteria for OMUES manual use ICU, intensive care unit; CCU, cardiac care unit; HCU, high care unit; ECU, environmental control unit; COVID-19, coronavirus disease 2019

Criteria for OMUES manual use
Inclusion criteria
≥15 years old
Japan Coma Scale (JCS) grade I
Capable of responding to questionnaires (no dementia, delirium, etc.)
Permitted for oral intake
Permitted for head-side up
Has neck flexibility
Exclusion criteria
Japan Coma Scale (JCS) grade II or higher
Incapable of maintaining seated and reclined positions. Postural adjustment permitted.
Incapable of holding the neck steady. Postural adjustment permitted.
In the terminal stage
Admitted to a high-intensity care unit (ICU, CCU, HCU, or ECU) or emergency ward
Admitted to the Department of Obstetrics
Hospital stay of two nights or less
Tested positive for COVID-19, has been in close contact with a patient with COVID-19 or met the COVID-19 triage conditions, and had a test result that cannot be confirmed to be negative.

**Figure 1:**
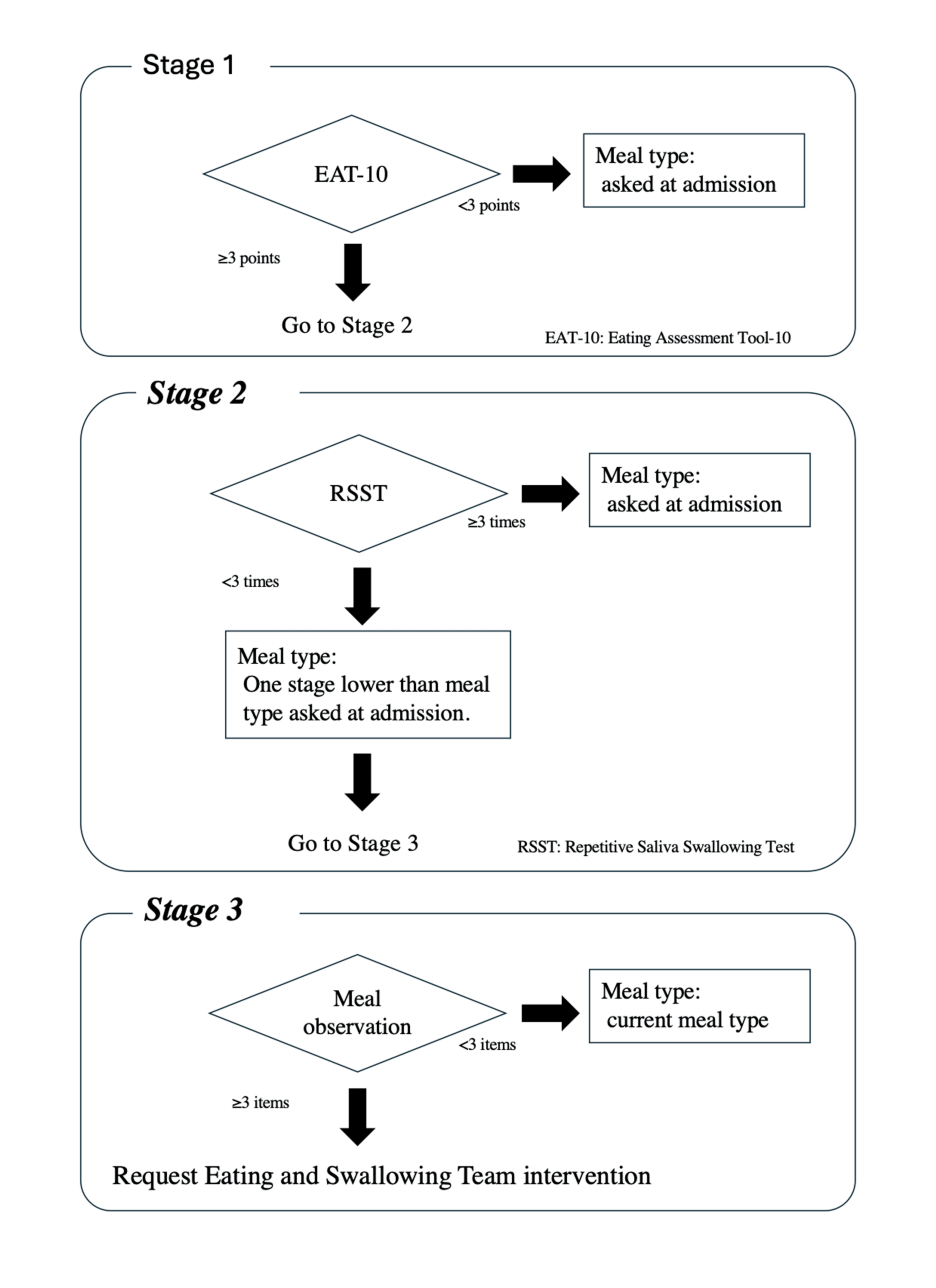
OMUES manual flowchart EAT-10, Eating Assessment Tool-10; RSST, Repetitive Saliva Swallowing Test

Patients who met the conditions for the use of the manual underwent a swallowing function assessment according to the flow chart in Figure 1. This flow chart is broadly divided into three stages. In stage 1, an eating questionnaire, the EAT-10, was administered. In stage 2, patients who scored ≥3 points on the EAT-10 underwent the RSST. In stage 3, patients who could not swallow ≥3 times during the RSST were observed while eating. When the assessment revealed problems in ≥3 of these items, the user was guided to request for EST intervention.

The EAT-10, a self-reported questionnaire used to assess swallowing function, was developed in 2008 by Belafsky et al. [12]. It consists of 10 items, each with a score of 0-4 points, and a total score of ≥3 points indicates a problem with swallowing function. The Japanese version of the EAT-10 questionnaire, which was translated and validated by Wakabayashi et al., is used in our hospital [13].

The RSST is a swallowing function screening test developed in 2000 by Oguchi et al., which indicates a high probability of dysphagia if the patient cannot swallow ≥3 times in a 30-s period [14,15]. It can be conducted by two suggested methods: swallowing simulated saliva and dry swallowing. In our hospital, we use the dry swallowing method.

In stage 1, the cutoff value is an EAT-10 score of three points, in line with the original study, with patients scoring <3 points assessed as having no swallowing risk. These patients or their families are asked about their usual meal type at admission and are started on an oral diet accordingly. Meal types are categorized in accordance with the Japanese Dysphagia Diet 2013 [16,17].

Patients who scored ≥3 points on the EAT-10 in stage 1 proceeded to stage 2 and underwent the RSST. The cutoff value for the RSST is swallowing three times, irrespective of age, with patients able to swallow ≥3 times assessed as having no swallowing risk. These patients or their families were asked about their usual meal type at admission and were started on an oral diet accordingly.

Patients who could not swallow ≥3 times in the RSST in stage 2 proceeded to stage 3. They were served meals of a type that was one stage lower than their current meal type and were observed while eating and assessed using the items listed in Table 2. When the assessment revealed problems in ≥3 of these items, an assessment was made by a certified dysphagia nursing nurse and an intervention proposed by the EST was initiated.

**Table 2:** Meal observation items

Item
Rounded back
Fails to eat slowly
Unable to transfer food smoothly from the mouth to the throat
Swallows repeatedly
Some food remains in the mouth after swallowing
Food is retained in the mouth
Takes time to swallow
Continues to chew without swallowing
Looks up to transfer food downwards
Dribbles food from the mouth
When asked to say “Ah” during or after the meal, voice quality is wet-hoarse (gurgling)
Coughs while eating

Patient characteristics

To identify the study participants, we used the data warehouse software Fujitsu HOPE/DWH-GX (Fujitsu Limited, Kawasaki, Japan) incorporated in the Fujitsu HOPE/EGMAIN-GX electronic medical records system that is used at our hospital. The search parameters comprised age, sex, admission date, length of hospital stay, primary disease, other conditions, and the results of the assessments included in the OMUES manual (EAT-10, RSST, and meal observation). The inclusion and exclusion criteria were in accordance with those in the OMUES manual shown in Table 1.

We reviewed the electronic medical records of patients documented as having aspiration pneumonia or suspected of having aspiration pneumonia and defined patients with aspiration pneumonia as those diagnosed with this condition by their primary clinical department based on chest radiography or chest computed tomography (CT) findings.

According to our hospital’s electronic medical records, 18,688 patients were admitted between September 1, 2021 and August 31, 2022. Of these, patients who participated in the OMUES manual trial (n=74), those who did not meet the inclusion criteria (n=2,591), and those who met the exclusion criteria (n=3,584) were excluded, leaving 12,439 patients. Furthermore, patients who were determined to have already been diagnosed with aspiration pneumonia on admission (n=44) were excluded, leaving 12,395 patients in this study.

**Figure 2:**
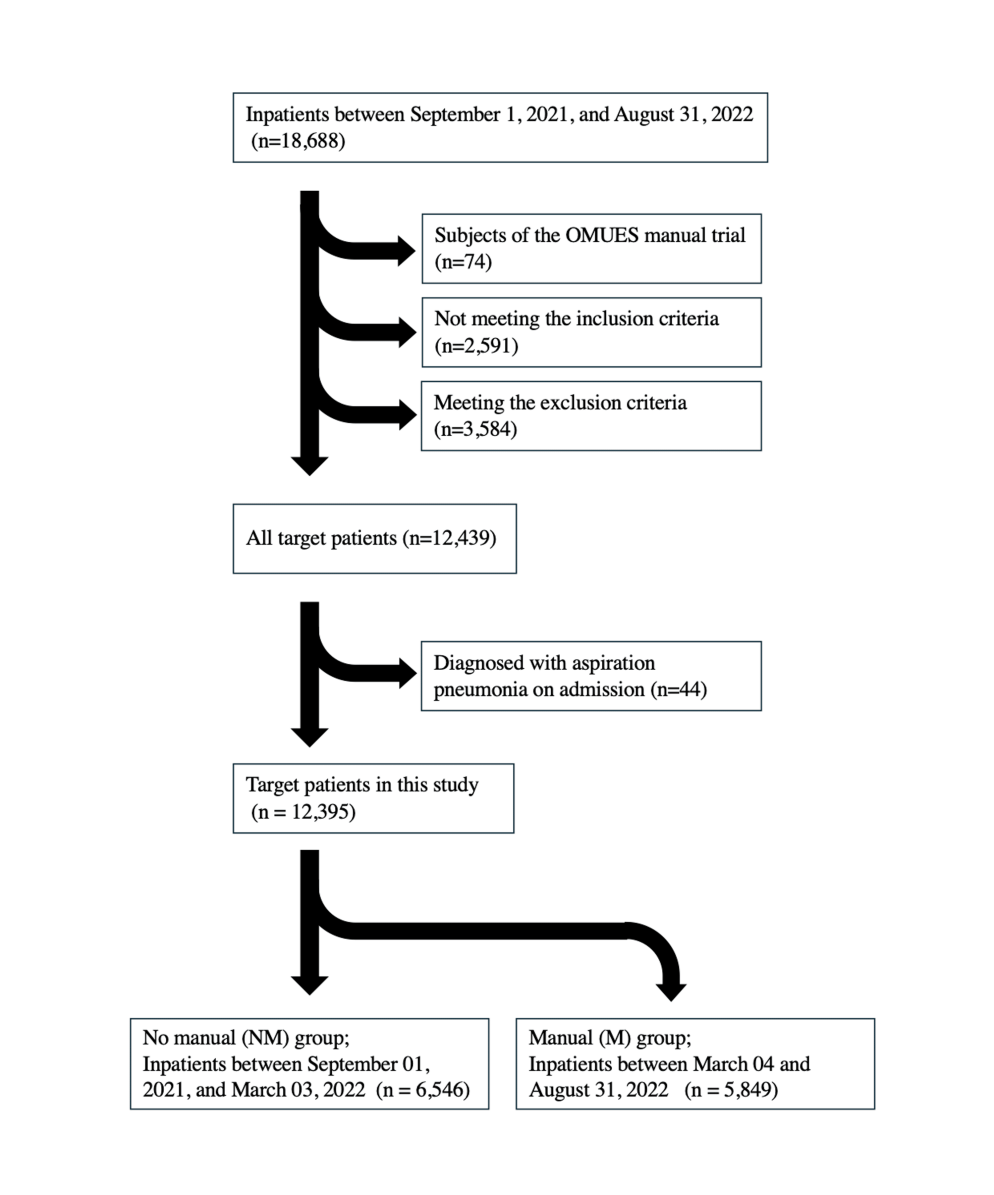
Flowchart of patient selection

The patients were divided into two groups: those admitted between September 1, 2021, and March 3, 2022 (before the OMUES manual was introduced) were classified as the no manual (NM) group (n = 6,546), while those admitted between March 4 and August 31, 2022 (after its introduction), were classified as the manual (M) group (n = 5,849) (Figure 2). There was no statistically significant difference in age, sex, or occurrence of aspiration pneumonia between the two groups.

We compared the number of cases of aspiration pneumonia between the NM and M groups. We also closely examined the electronic medical records of patients with aspiration pneumonia in order to identify the cause and compared the causes between the two groups.

The environment and circumstances at the time of aspiration pneumonia onset were identified from nursing and doctor records, and the causes of aspiration pneumonia were classified into seven categories: poor arousal (onset during hospitalization), iatrogenic (i.e. endoscopy, post-surgery, post-extubation, or other treatment), neuromuscular disease, pharyngolaryngeal/upper gastrointestinal disease, vomiting, respiratory disease, and other causes. As a secondary analysis, we also evaluated the status of the use of the OMUES manual in the M group, as well as its effects on the prevention of aspiration pneumonia.

Data analysis

For the two-group comparison, an unpaired t-test and Fisher's exact test were performed, and a p-value of <0.05 was considered to indicate statistical significance. Logistic regression analysis of the entire patient population, with aspiration pneumonia as the dependent variable, and age, sex, and OMUES manual usage as explanatory variables, was used for factor evaluation.

Logistic regression analysis of the M group who underwent the EAT-10 assessment, with aspiration pneumonia as the target variable, and age, sex, and EAT-10 score (<3 points or ≥3 points) as explanatory variables, was used for factor evaluation.

The statistical calculation software R for Mac OS X Cocoa GUI version 3.6.2 (R Foundation for Statistical Computing, Vienna, Austria) was used to perform the analysis [18].

Ethical considerations

Written informed consent could not be obtained because of the constraints imposed by the retrospective design. Participants could withdraw from this study at any time using an opt-out procedure. This study was reviewed and approved by the Ethics Committee of Osaka Metropolitan University Graduate School of Medicine (2022-146). The study did not require a waiver by virtue of its study design.

## Results

Of the 12,395 patients included in this study, 94 (0.8%) developed aspiration pneumonia, including 70 males and 24 females with a median age of 73.5 (interquartile range [IQR]: 15; range: 32-91) years and a median hospital stay of 24 (IQR: 28.75; range: 4-100) days. The 12,301 patients without aspiration pneumonia comprised 5,930 males and 6,371 females, with a median age of 67 (IQR: 23; range: 15-99) years and a median hospital stay of 10 (IQR: 9; range: 4-202) days. The patients with aspiration pneumonia were significantly older, included a larger proportion of males, and stayed in the hospital for longer.

There were 52 (0.8%) patients and 42 (0.7%) patients in the NM and M groups, respectively, with a non-significant difference (Table 3). There were no statistically significant differences between the two groups in terms of sex. The M group had a significantly lower number of patients whose cause of aspiration pneumonia was poor arousal than the NM group. Iatrogenic causes accounted for the largest number of patients in both the NM and M groups. Most aspiration pneumonia cases were caused by upper gastrointestinal endoscopy, which accounted for 13/21 (61.9%) cases in the NM group and 12/19 (63.2%) cases in the M group. All these patients developed aspiration pneumonia despite prophylactic antibiotic use.

**Table 3:** Patient characteristics NM group, no manual group; M group, manual group; IQR, interquartile range

Variable	NM group (n = 6,546)	M group (n = 5,849)	Total (n = 12,395)
Male	3,164	2,836	6,000
Female	3,383	3,013	6,395
Age (years)	67 (IQR: 53–76; range: 15–99)	67 (IQR: 53–76; range: 15–94)	67 (IQR: 53–76; range: 15–99)
Hospital stay	10 (IQR: 7–17; range: 4–202)	9 (IQR: 6–15; range: 4-155)	10 (IQR: 7–16; range: 4–202)
Aspiration pneumonia	52	42	94
Male (aspiration pneumonia)	39	31	70
Age (aspiration pneumonia)	74 (IQR: 65–80; range: 36–87)	73 (IQR: 67–79; range: 32–91)	73.5 (IQR: 66.25–80; range: 32–91)
Hospital stay (aspiration pneumonia)	22 (IQR: 10–38.25; range: 4–100)	25 (IQR: 14.25–40.25; range: 8–99)	24 (IQR: 12.25–38.75; range: 4–100)
Causes of aspiration pneumonia			
Poor arousal	12	1	13
Iatrogenic	21	19	40
Neuromuscular disease	5	5	10
Pharyngolaryngeal/upper gastrointestinal disease	3	8	11
Vomiting	6	3	9
Respiratory disease	3	4	7
Other	2	2	4

Evaluation of the OMUES manual use

In the M group, 5,412/5,849 (92.5%) patients underwent an aspiration risk assessment using the EAT-10 (stage 1), 176/346 (50.9%) patients with an EAT-10 score of ≥3 points were evaluated using the RSST (stage 2) of the OMUES manual, and 6/10 (60.0%) patients who were unable to swallow at least three times in the RSST were evaluated using stage 3 of the OMUES manual. The mean EAT-10 score was 0.9 ± 3.7 (0-40) points, and the median score was 0 points (IQR: 0; range: 0-40). In the RSST, the mean number of swallows was 4.7 ± 2.1 (0-15), and the median score was 4 points (IQR: 2; range: 0-15). The OMUES manual utilization rate, calculated by multiplying the utilization rates at each of stages 1, 2, and 3, was 28.3%.

A breakdown of each stage of the OMUES manual is shown in Table 4 and Table 5. Among patients who underwent EAT-10 evaluation, the incidence of aspiration pneumonia was 0.6% (34/5,412 patients), and these patients were significantly older, included a larger proportion of males, and tended to have stayed in the hospital for longer. Patients who scored ≥3 points on the EAT-10 accounted for 7/34 (20.6%) of those with aspiration pneumonia and 339/5,378 (6.3%) of those without aspiration pneumonia, with a significantly larger proportion of patients with aspiration pneumonia scoring ≥3 points on the EAT-10. Among patients who underwent RSST assessment, those with aspiration pneumonia were 7/176 (4.0%), and although they were significantly older than the patients without aspiration pneumonia, there was no significant difference in sex or length of hospital stay between groups. Only 1/7 (14.3%) of the patients among those with aspiration pneumonia and 9/169 (5.3%) of those without aspiration pneumonia were unable to swallow at least three times in the RSST, with a difference that was not statistically significant.

**Table 4:** Breakdown of stage 1 (EAT-10) For men and ≥3 points, a statistically significant difference was observed between aspiration pneumonia and no aspiration pneumonia using Fisher's exact test (p < 0.05). For age and hospital stay, a statistically significant difference was observed between aspiration pneumonia and no aspiration pneumonia using t-test (p < 0.05). EAT-10, Eating Assessment Tool-10

Items	Total (n)	Male	Female	Age (years)	Hospital stay	EAT-10 (≥3 points)	EAT-10 (<3 points)
Aspiration pneumonia	34	25	9	73 (IQR: 69.25–78.75; range: 34–87)	25 (IQR: 13.25–40.25; range: 9–99)	7 (20.6%)	27 (79.4%)
No aspiration pneumonia	5,378	2,594	2,784	67 (IQR: 53–76; range: 15–94)	9 (IQR: 6–15; range: 4–155)	339 (6.3%)	5,039 (93.7%)

**Table 5:** Breakdown of stage 2 (RSST) For men and <3 times, no statistically significant difference was observed between aspiration pneumonia and no aspiration pneumonia using Fisher's exact test. For hospital stay, no statistically significant difference was observed between aspiration pneumonia and no aspiration pneumonia groups using t-test. For age, a statistically significant difference was observed between aspiration pneumonia and no aspiration pneumonia groups using t-test (p < 0.05). RSST, Repetitive Saliva Swallowing Test

Items	Total	Male	Female	Age (years)	Hospital stay	RSST (<3 times)	RSST (≥3 times)
Aspiration pneumonia	7	6	1	76(IQR: 73–80.5 range: 67–85)	24(IQR: 21–33.5 range: 17–71)	1 (14.3%)	6 (85.7%)
No aspiration pneumonia	169	88	81	70(IQR: 60–75 range: 16–88)	10(IQR: 8–18 range: 4–155)	9 (5.3%)	160 (94.7%)

Table 6 shows a breakdown of the causes of aspiration pneumonia. Among patients who underwent EAT-10 evaluation, of the 34 patients with aspiration pneumonia, 27 had been assessed as not being at risk of aspiration, and the iatrogenic cases involved 18/27 (66.7%) of these patients. Among patients who underwent RSST assessment, of the seven patients with aspiration pneumonia, with the exception of the one patient with cerebrovascular disturbance, the other six had been assessed as not being at risk of aspiration, and in 3/6 (50.0%) of these patients, the cause was iatrogenic.

**Table 6:** Breakdown of cause of aspiration pneumonia EAT-10, Eating Assessment Tool-10; IQR, interquartile range; RSST, Repetitive Saliva Swallowing Test

Causes	EAT-10 (<3 points) (n = 27)	EAT-10 (≥3 points) (n = 7)	RSST (≥3 times) (n = 6)	RSST (<3 times) (n = 1)
Poor arousal	1	0	0	0
Iatrogenic	18	3	3	0
Vomiting	2	0	0	0
Neurological disease	2	3	2	1
Respiratory disease	2	1	1	0
Other	2	0	0	0

In all patients who proceeded to stage 3, problems were noted in fewer than three items during meal observation, oral ingestion was continued, and EST intervention was not required. In three patients, although intervention by EST was not regarded as necessary according to the manual, an ST intervention was nevertheless requested by the attending physician. One of these patients developed aspiration pneumonia.

Factorial analysis

A factorial analysis was conducted by performing logistic regression analysis of the entire patient population, with aspiration pneumonia as the target variable, and age, sex, and OMUES manual usage as explanatory variables. The results showed that there were significant positive correlations with age and the male sex but no significant difference in OMUES manual usage was evident (Table 7).

**Table 7:** The entire patient population Target variable was aspiration pneumonia. Explanatory variables were age, sex, and OMUES manual usage.

Items	B value	95% CI	95% CI lower	95% CI upper	p-value
(Intercept)	-8.129	1.274	-9.403	-6.855	0.000
Age	0.039	0.017	0.022	0.056	0.000
Sex (male)	1.064	0.466	0.598	1.529	0.000
Using the OMUES manual	-0.110	0.409	-0.519	0.299	0.599

As a secondary analysis, we also performed a logistic regression analysis on the M group who underwent the EAT-10 assessment, with aspiration pneumonia as the target variable, and age, sex, and EAT-10 score (<3 points or ≥3 points) as explanatory variables. The results revealed significant positive correlations with age, the male sex, and EAT-10 score of ≥3 points (Table 8).

**Table 8:** The members of the manual group who underwent the EAT-10 assessment Target variable was aspiration pneumonia. Explanatory variable were age, sex, and EAT-10 score (<3 points or ≥3 points). CI, confidence interval; EAT-10, Eating Assessment Tool-10

Item	B value	95% CI	95% CI lower	95% CI upper	p-value
(Intercept)	-8.808	2.230	-11.038	-6.579	0.000
Age	0.044	0.030	0.014	0.073	0.004
Sex (male)	0.985	0.766	0.219	1.751	0.012
EAT-10 scores	1.184	0.844	0.340	2.028	0.006

## Discussion

The OMUES manual is intended for dysphagia triage and leads to early EST intervention as a triage outcome, helping to prevent aspiration pneumonia. In this study, we investigated the effectiveness of the OMUES manual by investigating changes in the incidence of aspiration pneumonia.

In this study, we found that after introducing the OMUES manual, there was a significant decrease in the occurrence of aspiration pneumonia in patients with poor arousal. The OMUES manual is designed with assessments to be completed by the patients themselves and, therefore, cannot be used by patients with poor arousal on admission. In patients with poor arousal, the onset of aspiration pneumonia was during hospitalization; thus, they were included in the study. Since poor arousal developed after dysphagia triage using the OMUES manual, there is little reason to expect that the introduction of the manual would lead to a reduction in the incidence of aspiration pneumonia. Nevertheless, the number of patients with poor arousal declined after the manual was introduced. This may have been because the introduction of the manual had a secondary effect of raising awareness of aspiration risk among medical professionals, particularly nurses. This suggests that the OMUES manual may also have educational value, which is important from the perspective of medical safety.

There was no significant change in the incidence of aspiration pneumonia, which was 0.8% before and 0.7% after the manual’s introduction. One reason for these results might be the rate of utilization of the OMUES manual. Hinchey et al. investigated the status of compliance with swallowing screening in cerebral stroke patients in 15 institutions. They found that the compliance rate was 61%, but in institutions that conducted formal dysphagia screening, this rate was 78% compared with 57% in those that did not perform formal screening. The incidence of pneumonia was 2.4% in institutions that conducted formal screening and 5.4% in those that did not, a difference that was statistically significant [19]. The utilization rate of the OMUES manual was only 28.3%, which was lower than the rates in this previous report. However, a secondary analysis of patients in whom the OMUES manual was used found a positive correlation between EAT-10 assessment in stage 1 and aspiration pneumonia, suggesting that the low utilization rate of stages 2 and 3 of the OMUES manual after its introduction affected the incidence of aspiration pneumonia. The low implementation rate of the OMUES manual may have delayed professional intervention, which may have had no effect on the incidence of aspiration pneumonia.

Another possible cause is that patients with iatrogenic aspiration pneumonia would have had normal swallowing function at admission. In the majority of patients, iatrogenic aspiration pneumonia was associated with upper gastrointestinal endoscopy and occurred despite prophylactic antibiotic use, leading to the suspicion of chemical pneumonitis due to vomiting during endoscopic procedures. In most of these patients, swallowing function at admission was normal or only mildly impaired; therefore, these patients would not have been identified by using the OMUES manual at admission. As a result, its use was ineffective in reducing the incidence of aspiration pneumonia. Thus, an analysis including the primary disease at admission and type of surgery or treatment as additional assessment items might demonstrate the effectiveness of the OMUES manual.

One limitation of this study was that aspiration pneumonia was not clearly defined in our hospital. In this study, patients with aspiration pneumonia were defined as those diagnosed with this condition by their primary clinical department based on chest radiography or chest CT findings. If a different definition of aspiration pneumonia had been used, it is possible that more or less patients might have been included. For example, in Japan, the definition of aspiration pneumonia was established by the Japanese Respiratory Society [20,21], but this definition was not known to any clinical department or ward other than the respiratory medicine department in our hospital. If this definition had been used, the incidence of aspiration pneumonia would likely have been lower. This issue can be addressed by adopting uniform standards throughout the hospital. However, each department at our hospital has a high level of specialization, and thus we would like to continue raising awareness in the future.

A second limitation was that we did not verify the risks and preventive measures for aspiration pneumonia other than dysphagia. Different clinical departments each implement their own preventive measures, such as oral care interventions and antibiotic prophylaxis, and we did not address this aspect in this study. We also did not examine factors, such as rehabilitation, guidance on self-training using breathing training devices or patients’ activity levels and efforts to maintain them. Several reports exist on the usefulness of oral care and rehabilitation for preventing aspiration pneumonia [22-25]. In addition, Langmore reported that although dysphagia is an important risk factor for aspiration pneumonia, it is not a sufficient risk factor unless other factors are present [26]. All of these may affect the development of aspiration pneumonia, and further research is required. ESTs are currently active in all wards, and risk assessments and prevention methods will be standardized within our hospital.

Third, there is a possibility of selection bias in the patient population. Cerebral stroke patients are at high risk of aspiration pneumonia, and, as previously mentioned, they undergo a variety of swallowing screening tests. However, as emergency patients and patients who are unable to answer for themselves are excluded from the OMUES manual, the majority of cerebral stroke patients were not covered. Additionally, weight, height, and body mass index were not among the assessment items, which might have given rise to bias. These were initially included among the items investigated but were ultimately omitted because of the large amount of missing data. We also considered retaining these items and excluding the patients with missing data. In our electronic medical records system, weight and height are only reflected if they have been input in the designated fields in the electronic medical record. When these items are missing, it might be because of human error, such as forgetting to input this information; however, we were concerned that individual departments and wards have their own rules for managing weight and height data other than inputting them in the designated fields. If this were the case, excluding patients with missing data would be equivalent to excluding patients from specific clinical departments, resulting in a major bias; therefore, we decided to exclude these items instead.

Fourth, the assessment items did not include data, such as primary disease at admission, treatments provided, and medication. The Fujitsu DWH software used in this study is unable to distinguish primary and secondary diseases and identify items, such as treatments and medication.

To address the third and fourth limitations, we plan to solve these problems in the future by switching the data source to the Diagnosis Procedure Combination/Per-Diem Payment System.

## Conclusions

We investigated the effectiveness of the OMUES manual for dysphagia triage on all hospitalized patients by assessing changes in the incidence of aspiration pneumonia after introducing the OMUES manual. Although the use of the manual did not decrease the incidence of aspiration pneumonia, our results suggest that it is contributing to its prevention. In particular, the decrease in the incidence in patients with poor arousal suggests the possibility of an educational effect inducing a change in the nurses' awareness.

Its use has identified several problems that need to be addressed, including low implementation rates, the need to investigate availability of data, including data on the primary disease at admission, treatments provided, and medications, and the need to evaluate the effectiveness of rehabilitation. In addition, endoscopic examination revealed cases of aspiration pneumonia even in patients with normal swallowing function, and further investigation is required. In future, we plan to revise the OMUES manual, raise awareness within the hospital, and strive to triage patients with swallowing disorders, which is a risk factor for aspiration pneumonia.
